# Association between Precipitation Upstream of a Drinking Water Utility and Nurse Advice Calls Relating to Acute Gastrointestinal Illnesses

**DOI:** 10.1371/journal.pone.0069918

**Published:** 2013-07-16

**Authors:** Andreas Tornevi, Gösta Axelsson, Bertil Forsberg

**Affiliations:** 1 Occupational and Environmental Medicine, Department of Public Health and Clinical Medicine, Umeå University, Umeå, Sweden; 2 Occupational and Environmental Medicine, Department of Public Health and Community Medicine, University of Gothenburg, Gothenburg, Sweden; Unidad de Microbiología, Facultad de Medicina Universidad Rovira, Spain

## Abstract

**Background:**

The River Göta Älv is a source of fresh-water for the City of Gothenburg (Sweden). We recently identified a clear association between upstream precipitation and indicator bacteria concentrations in the river water outside the intake to the drinking water utility. This study aimed to determine if variation in the incidence of acute gastrointestinal illnesses is associated with upstream precipitation.

**Methods:**

We acquired data, covering 1494 days, on the daily number of telephone calls to the nurse advice line from citizens in Gothenburg living in areas with Göta Älv as a fresh-water supply. We separated calls relating to gastrointestinal illnesses from other medical concerns, and analyzed their association with precipitation using a distributed lag non-linear Poisson regression model, adjusting for seasonal patterns and covariates. We used a 0–21-day lag period for precipitation to account for drinking water delivery times and incubation periods of waterborne pathogens.

**Results:**

The study period contained 25,659 nurse advice calls relating to gastrointestinal illnesses. Heavy rainfall was associated with increased calls the same day and around 5–6 days later. Consecutive days of wet weather were also found to be associated with an increase in the daily number of gastrointestinal concerns. No associations were identified between precipitation and nurse advice calls relating to other medical concerns.

**Conclusion:**

An increase in nurse advice calls relating to gastrointestinal illnesses around 5–6 days after heavy rainfall is consistent with a hypothesis that the cause could be related to drinking water due to insufficient barriers in the drinking water production, suggesting the need for improved drinking water treatment.

## Introduction

Climate models project that Sweden will experience, along with a generally warmer and wetter climate, more extreme temperatures and more intense precipitation. According to the Swedish Climate and Vulnerability Assessment Report, increased precipitation will have a direct impact on drinking water quality [1]. More recurrent and intense precipitation increases run-offs from agricultural and urban areas and causes additional events of sewage release from overflowing combined sewer systems. This implies that more pathogens will enter raw water supplies, making it more challenging for drinking water utilities to produce safe drinking water. Ongoing climate change will increase microbiological risks primarily in surface water sources, because surface water shows greater and more rapid variations in quality compared to ground water [2].

High and low rainfall has previously been linked to gastrointestinal (GI) illnesses in various parts of the industrialized world [3]–[5]. The majority of studies on GI health have focused on outbreak situations, even though observed outbreaks are likely to represent only a fraction of the GI symptoms caused by poor drinking water [6], and the background burden remains unknown. However, some studies showing patterns in daily cases of GI illnesses even during periods when no unusual incidence was registered, suggest the cause to be the drinking water [7]–[13].

The river Göta Älv is a municipal surface water source for the City of Gothenburg and is exposed to upstream run-offs from agricultural areas and occasionally by overflowing combined sewer systems [14]. We previously analyzed the relationship between daily upstream precipitation, water turbidity, and indicator bacteria at the river water intake to the drinking water utility at Alelyckan, and found clear positive associations peaking two days after rainfall (Tornevi et al. unpublished data).

This study aimed to analyze the relationship between daily variations in GI symptoms in the population of Gothenburg and the amount of rainfall upstream of the drinking water utility. We wanted to determine if sporadic cases of gastroenteritis, which are otherwise hidden within the normal endemic level, might be related to precipitation, and thus be linked indirectly to the quality of the river water used for drinking water production. The results could have important implications for drinking water quality and the potential need to invest in a more advanced water treatment process.

## Materials and Methods

### Ethical Statement

This research article is part of a research project named: Climate Change, Fresh Water Quality, Treatment and Distribution- Assessment of Microbial Risks from Health Studies. The project has been approved by The Regional Ethical Review Board – Division of Medical Research, Umeå, Sweden (Dnr: 2010-259-32M). The Regional Ethical Review Board waived the requirement for participant consent since this project only uses anonymised register data. This research was conducted in Sweden.

### Drinking water production in Gothenburg and target population

Gothenburg is the second largest city in Sweden with a population of around 500,000. It has two drinking water utilities and uses two surface water sources for its municipal drinking water production: the river Göta Älv and a lake system (Delsjön) located at a higher altitude. The lake system is subject to fewer variations in water quality compared with the river. The drinking water utility at Alelyckan (AWU) mainly takes its raw water directly from the river, and distributes drinking water mainly to the northern part of Gothenburg.

The water level in the lake system is maintained by constantly supplying it with river water via a tunnel. The river water intake at AWU is closed at times when the water quality in the river is determined to be inadequate for drinking water production. To maintain the drinking water distribution, the water is then taken back from the lake through the tunnel directly to the utility. Protocols of river water intake closures are for example when E. coli samples are found to exceed 400 MPN/100 ml; if specific contamination events upstream are reported [14], or when high turbidity is observed for more than 1 hour. Although the distribution networks of the two water utilities are interconnected, geographical and hydraulic modeling can estimate the proportion of drinking water that each house receives from the AWU.

Previous research found that precipitation causes variations in river water quality. Therefore, this study focused on individuals living in households that received drinking water from the AWU. We defined the delivery area as households receiving at least 95% of their water from the AWU alone, which accounted for about one third of the population of Gothenburg. The drinking water production at the AWU uses a conventional water treatment technique with barriers consisting of: chemical flocculation with alum and sedimentation, rapid filtration through granular activated carbon, and disinfection with chlorine/chlorine-dioxide. Production time is around 6–8 hours. The distribution time varies with demand and distance but is generally within one day for a majority of the households in the area.

### Health data

The telephone nurse advice line is a well-established and free medical service in Sweden. It is open 24-hours and is intended for non-emergency medical concerns. We obtained data on all phone calls to the nurse advice line from individuals residing in the AWU delivery area for the period from November 29, 2007, to December 31, 2011, including whether or not the call was associated with acute GI illnesses. GI concerns were defined based on three symptoms (vomiting, stomach pain, and diarrhea). Non-GI symptoms where uncategorized (other concern). Five dates were identified with incomplete data recording and were set as missing values.

### Meteorological data

The Swedish Meteorological and Hydrological Institute provided complete daily weather data from the Gothenburg area and upstream for Göta Älv. Based on previous analyses, we choose precipitation data from a meteorological station situated about 30 km upstream from the AWU (meteorological station Alvhem), as this station has powerful predictive abilities for turbidity and indicator bacteria outside the AWU water intake.

### Statistical analysis

We studied the relationship between daily precipitation and daily number of telephone calls to the nurse advice line relating to GI illnesses (vomiting + stomach pain + diarrhea) from individuals living within the AWU delivery area (henceforth referred as GI calls), using time-series regression. We assumed that GI calls followed a Poisson distribution, and modeled their association with precipitation using generalized additive regression [15]. To consider incubation time of water borne infections, drinking water production and distribution time, we analyzed GI calls with a 0–21-day lag period for precipitation. We designed a distributed lag non-linear model (DLNM) to allow associations to vary smoothly along dimensions of precipitation and lag days [16]. This model design is parameter efficient and can detect more than one association that might be present due to various incubation times along the predefined lag period. We adjusted for potential confounding variables and covariates: seasonality and time trend, holidays and days around holidays, day of week, and daily mean temperature. We also adjusted for daily number of nurse advice calls regarding non-GI symptoms, with the intention to purify any associations between GI symptoms and precipitation, and to adjust for daily variations in nurse advice call activity that other covariates could not explain.

A DLNM design assumes that the effects of exposure at each lag are independent. Therefore, to consider if periods (two days or more) of precipitation were related to daily variations of GI calls, we introduced an additional precipitation predictor for consecutive days of wet or dry weather.

An algebraic expression of a model could be written as:
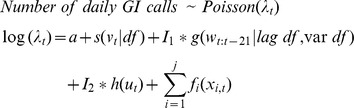
where *t* corresponds to each day in the study, and *s* is function represented by a thin-plate-spline that simultaneously adjusts for seasonal patterns and time trend. Hence, *v* represents an ordered discrete count of the observation days. The flexibility parameter was set to ∼7 degrees of freedom (df) per year. An illustration of a smooth seasonal time trend function is presented in Figure S1. The function *g* is two-dimensional and defines the distributed lag non-linear predictor of daily precipitation (*w*) for lags 0–21, and is controlled by independent parameters in each dimension (*lag df*, and *var df*). The function *h* represents an association with number of consecutive days of dry or wet weather (*u_t_*), where a wet-weather day was defined by *w*>0. We modeled this predictor as a categorical variable that was truncated to hold at least 30 unique consecutive wet or dry periods in each category, resulting in eleven levels (>5 dry days, 5th dry, 4th dry, 4th wet, >4 wet days). These precipitation predictors, the DLNM predictor (*g*) and the consecutive days of wet or dry weather predictor (*h*), were fitted separately ({*I*_1_ = 1, *I*_2_ = 0} and {*I*_1_ = 0, *I*_2_ = 1}) and jointly ({*I*_1_ = 1, *I*_2_ = 1}) to examine modifications attributable to collinearity. Finally, *x_i_* denotes all other covariates described by functions *f_i_*, where calendar factors were parameterized as indicator variables while others as continuous variables. Continuous covariates were modeled to associate with GI calls linearly or alternatively non-linearly with a thin-plate-spline. The covariate representing nurse advice calls regarding non-GI symptoms where before inclusion adjusted for seasonality, time trend and day of week, to make daily variations comparable through the whole time series. We kept covariates in the model if they showed to hold a predictive ability to the outcome.

Various DLNM designs were tested and we obtained the best parameter setting (lag df, var df) by systematic comparison of various numbers of lag knots and distances between internal lag knots. We let Akaike Information Criterion (AIC) choose a best design for the model.

We also validated if the modeled associations could be considered robust with respect to the seasonal time-trend component by relaxing or tighten this components elasticity from 3 to 12 df per year and compared differences in estimated effects.

As a complementary analysis, using similar assumptions and model design, we also studied non-GI symptoms as a model outcome. The intention was to validate if the predictors of GI symptoms could be considered unique for this type of medical concern.

All statistical analyses were carried out in R 2.11.1 [17], using MGCV [15] and DLNM [16] packages.

## Results

### Descriptive statistics

A total of 25,659 phone calls to the nurse advice line relating to GI illnesses were registered during the study period from individuals living in the AWU delivery area. There were 3 to 47 calls per day (Table 1) with a distinct seasonal pattern; the frequency of calls was higher during winter periods (Figure S1). Precipitation occurred on 46% of the days with a 24-h average of 3.2 mm, or 7.1 mm if only days with precipitation were analyzed (Figure S2, Table 1).

**Table 1:** Descriptive statistics on daily nurse advice calls and weather data.

	Percentiles, mean and standard deviation (SD)										
	Min	5th	10th	25th	50th	75th	90th	95th	Max	Mean	SD
Nurse advice calls											
GI symptoms	3	8	9	12	16	21	27	30	47	17.2	6.9
Non-GI symptoms	44	72	79	90	103	116	129	137	236	104.2	22.6
Weather											
Precipitation (mm)	0.0	0.0	0.0	0.0	0.0	4.1	10.7	16.2	47.3	3.2	6.1
Precipitation >0 (mm)	0.1	0.3	0.5	1.7	4.8	10	16.9	23.9	47.3	7.1	7.4
Consecutive dry days	1	1	1	1	3	5	10	14	38	4.5	5.4
Consecutive wet days	1	1	1	1	2	4	6	8	14	2.8	2.3
Temperature (C)	−12.7	−4.5	−1.3	3.0	8.4	14.8	17.9	19.7	25.4	8.4	7.6

### Effect of precipitation on nurse advice calls

**Figure 1:**
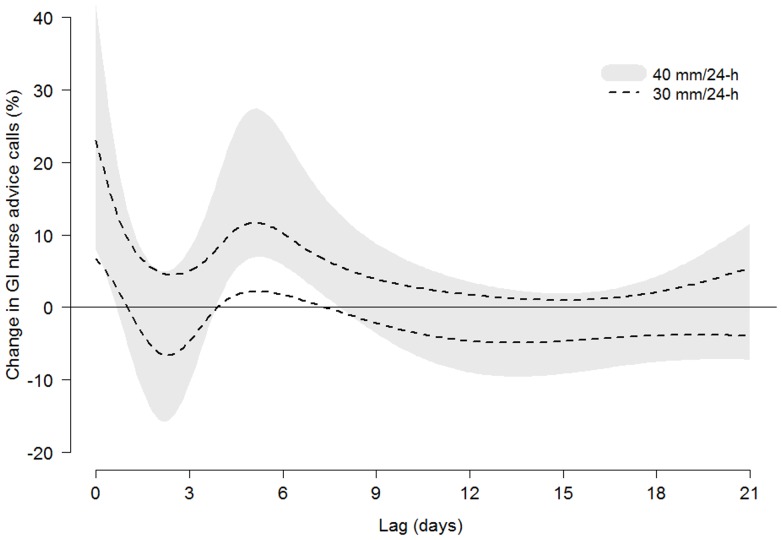
Effect of heavy precipitation on GI calls. Confident intervals (95%) of associations along 0–21 lag days between heavy precipitation events and nurse advice calls relating to gastrointestinal symptoms.

Regression models assessed an association between precipitation and GI calls within the first lag week. Heavy precipitation (>25 mm/24-h) was associated with an increase in GI calls on the same day and around 5–6 days later. A non-linear distributed lag model estimated an event of 30 mm/24-h with an increase in GI calls of: 15% at lag 0 (95% confident interval (CI): 6–23%), 7% at lag 5 (95% CI: 2–12%), and 6% at lag 6 (95% CI: 2–11%). Table 2 displays estimated effects of heavy rainfall at lags with significant associations. Figure 1 shows confidence intervals of modeled associations between precipitation and GI calls for events of 30 mm/24-h and 40 mm/24-h along lags 0–21.

**Table 2:** Associations between heavy precipitation and GI nurse advice calls (DLNM model).

	Percent change in GI calls (95% confident intervals)				
Precipitation (mm/24-h)	Lag 0	Lag 4	Lag 5	Lag 6	Lag 7
25	11 (5–16)	2 (-0–5)	3 (0–6)	3 (0–6)	2 (-0–4)
30	15 (7–23)	4 (0–9)	7 (2–12)	6 (2–10)	4 (1–7)
35	19 (8–32)	7 (1–13)	11 (4–19)	10 (4–16)	7 (2–12)
40	24 (8–42)	10 (2–19)	17 (7–27)	15 (6–24)	10 (3–17)
45	29 (8–54)	13 (2–25)	22 (9–37)	20 (8–32)	13 (4–23)

**Figure 2:**
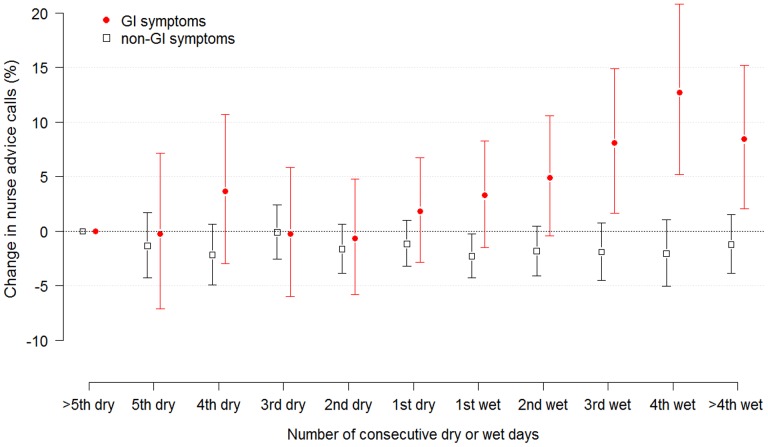
Effect of consecutive days with dry- or wet weather. Estimated change in daily number of nurse advice calls relating to GI symptoms and non-GI symptoms. Vertical bars represent 95% confidence intervals and ‘more than 5 consecutive dry days’ is selected as a reference category.

Days of wet weather increased the daily number of nurse advice calls relating to GI symptoms. An overall comparison between dry and wet days estimated an average increase in GI calls of 5% (95% CI: 3–8%), while considering consecutive wet days an increase of 13% (95% CI: 5–21%) was estimated on the fourth day of wet weather, compared to dry periods of 5 days or more (Figure 2).

No associations between precipitation and non-GI symptoms were identified when comparing dry and wet days (95% CI: -1.9–0.2%). A DLNM model with non-GI symptoms as an outcome did not detect any delayed effects, and no obvious patterns with consecutive wet or dry weather days were identified. Figure 2 displays the effect estimations of consecutive wet or dry days on nurse advice calls regarding GI symptoms and non-GI symptoms.

### Model details and sensitivity analysis

A DLNM design with a natural cubic spline used both in precipitation space and lag space described associations well, and was the least sensitive design with respect to parameter setting (lag df, var df). However, other non-linear designs, for example a polynomial spline, ended up with the same interpretation of results. We identified that 2–3 df in predictor space (1 or 2 internal knot) together with 5–6 df in lag space (3–4 internal knots with a lag knot window of 2.2–2,5 days) showed the best predictive DLNM parameter setting according to AIC (Figure S3). We report results from the model with the minimum number of parameters and absolute minimum AIC score: 2 df in predictor space (knot at 10 mm) and 5 df in lag space (lag knots at 2.2, 4.4, 6.6).

When modeling GI calls with both precipitation predictors simultaneously ({*I*_1_ = 1, *I*_2_ = 1}), the DLMN association at low– and moderate precipitation at lags 0–1 was relocated to the categorical predictor (Figure S4). Hence, the positive association with low– and moderate precipitation in early lags described by the DLNM predictor when {*I*_1_ = 1, *I*_2_ = 0} seams to originates from episodes of consecutive wet weather.

The delayed associations with heavy rainfall around lag 5–6 were similar when adding the consecutive weather day's predictor to the model (Figure S4). The effect estimations of consecutive dry or wet weather showed similar effect estimates when modeling this predictor separately ({*I*_1_ = 0, *I*_2_ = 1}) or jointly ({*I*_1_ = 1, *I*_2_ = 1}) with the DLNM predictor.

Covariates with predictive ability on GI calls were: day of week (Figure S5), national holiday, day before national holiday, and nurse advice calls regarding non-GI concerns. Associating non-GI calls non-linearly showed that the reason for this relation were days with an unusual low number of nurse advice calls. None of these covariates, or daily mean temperature, influenced the association between precipitation and GI-calls. Similar effects of calendar factors were observed when analyzing non-GI calls, although this outcome showed a more irregular seasonal pattern and peaked rapidly during a vaccination campaign against H1N1 influenza (November 2009).

All associations between precipitation and with GI calls showed robustness when adjusting the flexibility in the seasonal time-trend component (Figure S6). Diagnostic analysis of model residuals showed that they followed a normal distribution, and indicated that no serial correlation remained (Figure S7).

## Discussion

### Findings

An increase in nurse advice calls relating to GI illnesses around 5–6 days after heavy precipitation is consistent with a hypothesis that the cause can be drinking water related. In a previous study, we concluded that river raw water was most affected by rainfall two days prior, and it is reasonable to assume that drinking water production and delivery take place within a few days. If the observed increase in GI related nurse advice calls about 5–6 days after heavy rainfall is caused by pathogens in the drinking water, this suggests an illness with short incubation period. The peak at lag 5 would represent an incubation period of 1–2 days, assuming that medical advice was sought promptly.

Incubation times for gastroenteritis caused by waterborne pathogens vary widely depending on the infectious agent, and may also vary according to the infectious dose intake. Waterborne viruses such as the Norwalk agent have short incubation times (1–2 days), while protozoal infections such as cryptosporidiosis or giardiasis usually have longer and a wider range of incubation periods (giardiasis: 3–25 days, median 7–10 days; cryptosporidiosis: 2–12 days, median 7 days) [18]. The incubation period for bacterial infections also varies according to the agent, but the median period is usually within 0–5 days (campylobacter: range 1–10 days, median around 2–5 days) [18]. Infectious doses of viral and protozoal agents are lower than those for bacteria, and despite the fact that waterborne viral outbreaks have rarely been identified, Norwalk-like viruses are acknowledged as the major cause of waterborne illnesses worldwide [19]. Because viruses are also much smaller than bacteria, they are less likely to be removed during filtration, and typically have a greater resistance to disinfection than bacteria [20]. We therefore suggest that the observed delayed association around lag 5–6 were more likely due to contamination of drinking water by viral rather than bacterial or protozoal pathogens. The observed seasonality in GI calls, with the highest frequency in the beginning of the year, is also most likely a reflection of the seasonality in enteric virus infections.

It seems unlikely that the increase in GI nurse advice calls observed the same day as heavy precipitation could be related to drinking water due to poor quality of river water, because this would have been too shortly for microbiological contamination in the river water to have an influence on the incidence. Other studies, however, have suggested that old infrastructures can allow infiltration of drinking water supplies where they run in close proximity to sewage pipes, so that contamination of drinking water may occur after it leaves the drinking water utilities [21], [22]. Although heavy rainfall undoubtedly stresses the storm-water systems, there are no known instances of any such sewage intrusions into the drinking water delivery system within our study area.

Likewise, the reason for the increasing trend in GI calls with number of consecutive precipitation days is unclear. A speculative explanation, besides drinking water, could be that prolonged precipitation changes people's activity patterns, resulting in less outdoor activity, for instance in day-care centers, and possibly more people using public transport. These behavior patterns would increase the risk of transmission of infectious diseases. Another speculative explanation might involve a psychological effect similar to the observed weekday pattern, in which Mondays have the highest frequency of nurse advice calls and Fridays the lowest frequency (Figure S5). To the best of our knowledge, no studies have investigated the effects of weather on people's motivation to seek telephone medical advice. Conversely, some studies have shown an opposite effect regarding emergency department visits, with fewer visitors on wet days [23]. However, most studies that aimed at predicting daily numbers of patients found no or only modest effects of dry or wet weather [24].

### Other studies

The effect and lag structures of precipitation on fresh-water quality and drinking water production and distribution times can be assumed to vary widely between cities, making comparisons with other populations unreliable. Only a few studies investigated daily precipitation as an exposure variable in relation to daily variations in cases of GI problems under normal endemic incidence. A study from Milwaukee (WI) reported an increase of 11% in emergency department visits 4 days after any amount of rainfall [7].

Several studies reported associations between GI disease outbreaks and preceding precipitation. In an evaluation of 584 reported outbreaks (1948–1994) in the United States, the majority were preceded by heavy rainfall events [3]. In a study from England and Wales regarding 89 drinking water-related outbreaks (1910–1999), cumulative rainfall over 7 days was associated with increased risk [4], and accumulated rainfall over 5 days was significantly associated with waterborne disease outbreaks when investigating 92 events in Canada between the years 1975–2001 [5]. Cumulative daily rainfall has also been used as exposure variable in time series studies. In a large study on cryptosporidiosis in England with data between the years 1990–1999, seven days of rainfall in the North West region were shown to increase the overall weekly rate of laboratory confirmed cases, especially in areas where large seasonal patterns were observed [25].

Water turbidity has typically been used as an exposure predictor for daily variations in GI health in studies on periods of non-outbreak situations. Erogov et al. [8] reported a 1–2 day lag effect for self-reported GI symptoms, while Gilbert et al. [9] reported positive associations with the daily use of a health information telephone line, 11, 15 and 17 days later. An increase in pediatric GI visits to the emergency department in Philadelphia was observed 4 days after increased turbidity [12], and hospital admissions in the same area were also associated with increased turbidity in the elderly [11] and children [12], with a delayed effect. Cryptosporidiosis has also been associated with water turbidity. Morris et al. reported that increased levels of effluent water turbidity increased GI events in Milwaukee (WI) at lags that coincide with incubation time for this illness. These associations were observed when analyzing hospital visits and admissions during the year prior to, and the period within, the massive cryptosporidiosis outbreak that occurred in 1993 [26].

### Data limitations and advantages

Data on nurse advice calls do not include information on the diagnosis (e.g. ICD codes or stool analysis), so the cause of the illness cannot be determined. However, given that the medical conditions concerned generally did not require prescribed medication or hospital admission, these data have the advantage of capturing a higher proportion of the infected population compared to other types of individual-based health-data sources, such as emergency-room or doctor visits. Data that identify a higher proportion of the actual cases increase the probability of identifying relationships. The Swedish National Food Agency has conducted studies to detect the actions people take during acute gastroenteritis. National telephone interviews (n = 1000) found that about 9% said they would use the nurse advice line for medical guidance during gastroenteritis [27]; this proportion is believed to be many times higher than the proportion who seek personal medical advice or treatment. Another advantage of studying nurse advice calls as a population health marker is that they probably provide a more rapid and concentrated reflection of the population status for non-emergency symptoms.

Potential inaccuracies could arise if people called for medical advice whilst on holiday or temporary housing elsewhere, or if individuals, despite their registered home address, did not in reality live within the delivery zone. However, these potential sources of misclassification would not create the observed short-term associations. Also, it could be assumed that temporal demographic variations could occur, for example in relation with school holidays, but it is unlikely that such patterns would be the cause for the observed short-term relationships between GI calls and rainfall.

Secondly, this study did not take account of the fact that the AWU occasionally closes the river water intake when the water is considered too contaminated for drinking water production and instead takes water back through the tunnel, river water first and then water from the lake system. This regulation of raw water intake has been shown to be of great importance as a microbiological barrier [14], even though the substitute fresh-water supply is otherwise constantly supplied with river water.

### Conclusions

This study reports an association between precipitation events upstream of a drinking water utility and nurse advice calls related to GI illnesses. The observed increase around 5–6 days after heavy precipitation is consistent with our main hypothesis that the association results from poor fresh-water quality, and the lags suggest a primarily viral cause of contamination. Increases in GI symptoms with no lag period and following consecutive wet-weather days are harder to relate direct to the fresh-water quality and motivates further studies on this topic. These findings suggest that greater investment may be required in more advanced barriers in the drinking water production, with respect to public health impacts and possible greater risks due to effects of climate change.

## Supporting Information

Figure S1 **GI calls.** Daily number of nurse advice calls related to GI symptoms from individuals residing in the Alelyckan drinking water utility delivery zone from November 29, 2007 to December 31, 2011). A smooth spline (7 df per year) describes the seasonal patterns and trend (black curved line). Horizontal lines represent selected percentiles.
(TIFF)

Figure S2 **Precipitation.** Daily registered precipitation from November 08, 2007 to December 31, 2011, 30 km upstream of the raw water intake of the Alelyckan drinking water utility. Horizontal lines represent selected percentiles.
(TIFF)

Figure S3 **AIC scores.** AIC scores from models describing the association between precipitation and GI-calls with different knot settings in lag space in the DLNM predictor, with use of natural cubic spline design and one internal knot in predictor space. Colors represent the change in the AIC score when including the DLNM predictor in the model. Lower triangular area has invalid combinations and represents a model with zero internal lag knots. The AIC suggest that a best lag knot setting is 3 internal lag knots with a lag knot window at 2.2 ({2.2, 4.4, 6.6}).
(TIFF)

Figure S4 **Modifications due to collinearity.** Estimated change in nurse advice calls (%) relating to GI symptoms to daily precipitation (0–47 mm) along 0–21 lags with and without adjustment for consecutive dry or wet weather days. Red areas show where significant positive relationships are estimated in at least 2 consecutive days. A: a distributed non-linear lag model not adjusted for consecutive days with dry or wet weather. B: a distributed non-linear lag model adjusted for consecutive days of wet weather.
(TIFF)

Figure S5 **Day of week effect.** Effect of day of week on nurse advice calls where Mondays is selected as reference day.
(TIFF)

Figure S6 **Sensitivity analyzes.** Association between precipitation and GI calls with different settings (df) in the seasonal- trend component (10 models). Light gray (3 df per year) – dark gray (12 df per year). A: estimated change (%) in GI calls of an event of 35 mm/24-h of precipitation along 0–21 lag days. B: estimated change (%) in GI calls with consecutive dry or wet weather days.
(TIFF)

Figure S7 **Model residuals.** Diagnostic plots of model residuals (DLNM model with trend/season component using 7 df per year). A: time series residual plot indicating constant variance with time a no seasonal pattern. B: histogram and qq-plot show that residuals follow a normal distribution. C: the cumulative periodogram indicate no serial correlation in model residuals.
(TIFF)

## References

[1] Swedish Commission on Climate and Vulnerability (2007) Sweden facing climate change – threats and opportunities, Stockholm. Swedish Governmental Official Reports (SOU) 2007:60. Final report. Available in Swedish from: http://www.sweden.gov.se/sb/d/8704/a/89334 Sverige inför klimatförandringarna. Bilaga B 13: Dricksvattenförsörjning i förändrat klimat.

[2] Delpla I, Jung AV, Baures E, Clement M, Thomas O (2009) Impacts of climate change on surface water quality in relation to drinking water production. Environ Int 35: 1225–1233. 19640587 10.1016/j.envint.2009.07.001

[3] Curriero FC, Patz JA, Rose JB, Lele S (2001) The association between extreme precipitation and waterborne disease outbreaks in the United States, 1948–1994. Am J Public Health 91: 1194–1199. 11499103 10.2105/ajph.91.8.1194 PMC1446745

[4] Nichols G, Lane C, Asgari N, Verlander NQ, Charlett A (2009) Rainfall and outbreaks of drinking water related disease and in England and Wales. J Water Health 7: 1–8. 18957770 10.2166/wh.2009.143

[5] Thomas KM, Charron DF, Waltner-Toews D, Schuster C, Maarouf AR, et al (2006) A role of high impact weather events in waterborne disease outbreaks in Canada, 1975–2001. Int J Environ Health Res 16: 167–180. 16611562 10.1080/09603120600641326

[6] Reynolds KA, Mena KD, Gerba CP (2008) Risk of waterborne illness via drinking water in the United States. Rev Environ Contam Toxicol 192: 117–158. 18020305 10.1007/978-0-387-71724-1_4 PMC7120101

[7] Drayna P, McLellan SL, Simpson P, Li SH, Gorelick MH (2010) Association between Rainfall and Pediatric Emergency Department Visits for Acute Gastrointestinal Illness. Environmental Health Perspectives 118: 1439–1443. 20515725 10.1289/ehp.0901671 PMC2957926

[8] Egorov AI, Naumova EN, Tereschenko AA, Kislitsin VA, Ford TE (2003) Daily variations in effluent water turbidity and diarrhoeal illness in a Russian city. Int J Environ Health Res 13: 81–94. 12745350 10.1080/0960312021000071567

[9] Gilbert ML, Levallois P, Rodriguez MJ (2006) Use of a health information telephone line, Info-sante CLSC, for the surveillance of waterborne gastroenteritis. J Water Health 4: 225–232. 16813015

[10] Morris R, Naumova EN, Levin R, Munasinghe R (1996) Temporal variation in drinking water turbidity and diagnosed gastroenteritis in Milwaukee. American journal of public health 86: 237–239. 8633742 10.2105/ajph.86.2.237 PMC1380334

[11] Schwartz J, Levin R, Goldstein R (2000) Drinking water turbidity and gastrointestinal illness in the elderly of Philadelphia. J Epidemiol Community Health 54: 45–51. 10692962 10.1136/jech.54.1.45 PMC1731533

[12] Schwartz J, Levin R, Hodge K (1997) Drinking water turbidity and pediatric hospital use for gastrointestinal illness in Philadelphia. Epidemiology 8: 615–620. 9345659 10.1097/00001648-199710000-00001

[13] Tinker SC, Moe CL, Klein M, Flanders WD, Uber J, et al (2010) Drinking water turbidity and emergency department visits for gastrointestinal illness in Atlanta, 1993–2004. Journal of Exposure Science and Environmental Epidemiology 20: 19–28. 18941478 10.1038/jes.2008.68 PMC3752848

[14] Astrom J, Petterson S, Bergstedt O, Pettersson TJR, Stenstrom TA (2007) Evaluation of the microbial risk reduction due to selective closure of the raw water intake before drinking water treatment. Journal of Water and Health 5: 81–97. 17890838 10.2166/wh.2007.139

[15] Wood SN (2011) Fast stable restricted maximum likelihood and marginal likelihood estimation of semiparametric generalized linear models. Journal of the Royal Statistical Society Series B-Statistical Methodology 73: 3–36.

[16] Gasparrini A, Armstrong B, Kenward MG (2010) Distributed lag non-linear models. Stat Med 29: 2224–2234. 20812303 10.1002/sim.3940 PMC2998707

[17] Team R (2010) R: A Language and Environment for Statistical Computing. R Foundation for Statistical Computing, Vienna, Austria, 2007.ISBN 3-900051-07-0.

[18] Chin J (2000) Control of communicable diseases manual: American Public Health Association Washington, DC.

[19] Leclerc H, Schwartzbrod L, Dei-Cas E (2002) Microbial agents associated with waterborne diseases. Crit Rev Microbiol 28: 371–409. 12546197 10.1080/1040-840291046768

[20] Payment P (1999) Poor efficacy of residual chlorine disinfectant in drinking water to inactivate waterborne pathogens in distribution systems. Can J Microbiol 45: 709–715. 10528403

[21] Hunter PR, Colford JM, LeChevallier MW, Binder S, Berger PS (2001) Waterborne diseases. Emerg Infect Dis 7: 544. 11485661 10.3201/eid0707.017723 PMC2631838

[22] LeChevallier M, Gullick R, Karim M, Friedman M, Funk J (2003) The potential for health risks from intrusion of contaminants into the distribution system from pressure transients. J Water Health 1: 3–14. 15384268

[23] Diehl AK, Morris M, Mannis SA (1981) Use of calendar and weather data to predict walk-in attendance. Southern medical journal 74: 709. 7244751 10.1097/00007611-198106000-00020

[24] Wargon M, Guidet B, Hoang T, Hejblum G (2009) A systematic review of models for forecasting the number of emergency department visits. Emergency Medicine Journal 26: 395–399. 19465606 10.1136/emj.2008.062380

[25] Naumova EN, Christodouleas J, Hunter PR, Syed Q (2005) Effect of precipitation on seasonal variability in cryptosporidiosis recorded by the North West England surveillance system in 1990–1999. J Water Health 3: 185–196. 16075943

[26] Morris RD, Naumova EN, Griffiths JK (1998) Did Milwaukee experience waterborne cryptosporidiosis before the large documented outbreak in 1993? Epidemiology 9: 264–270. 9583417

[27] Toljander J, Karnehed N (2012) A descriptive study of behavioural patterns associated with gastrointestinal illness in the general Swedish population. Journal of Public Health and Epidemiology 4: 30–33.

