# Supplementary figures and images for: Association between Precipitation Upstream of a Drinking Water Utility and Nurse Advice Calls Relating to Acute Gastrointestinal Illnesses
Source: PLoS One. 2013 Jul 16;8(7):e69918. doi: 10.1371/journal.pone.0069918 (PMC3713056; doi:10.1371/journal.pone.0069918)

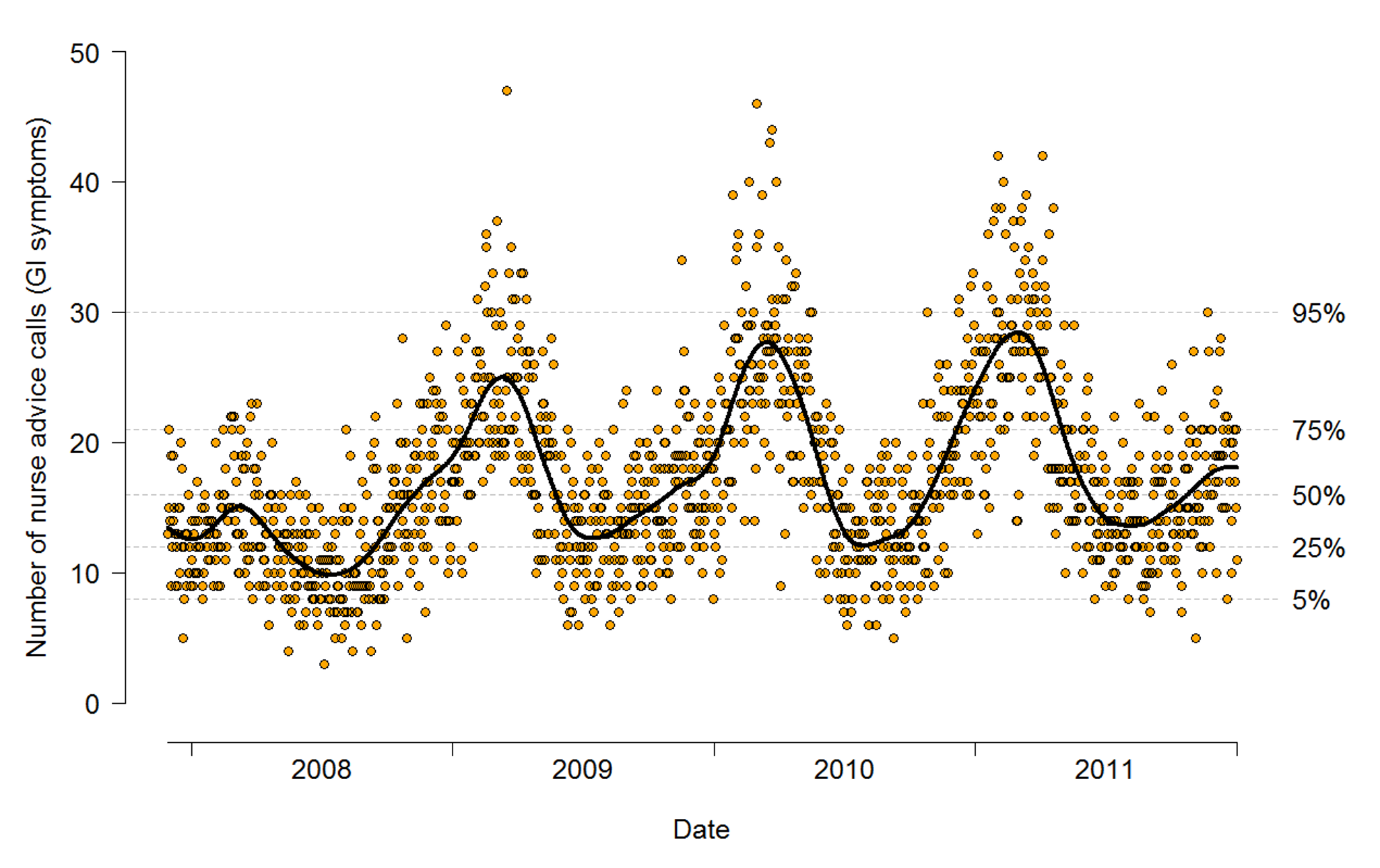

Supplement: Figure S1 — GI calls. Daily number of nurse advice calls related to GI symptoms from individuals residing in the Alelyckan drinking water utility delivery zone from November 29, 2007 to December 31, 2011). A smooth spline (7 df per year) describes the seasonal patterns and trend (black curved line). Horizontal lines represent selected percentiles. (TIFF) [file pone.0069918.s001.tif]

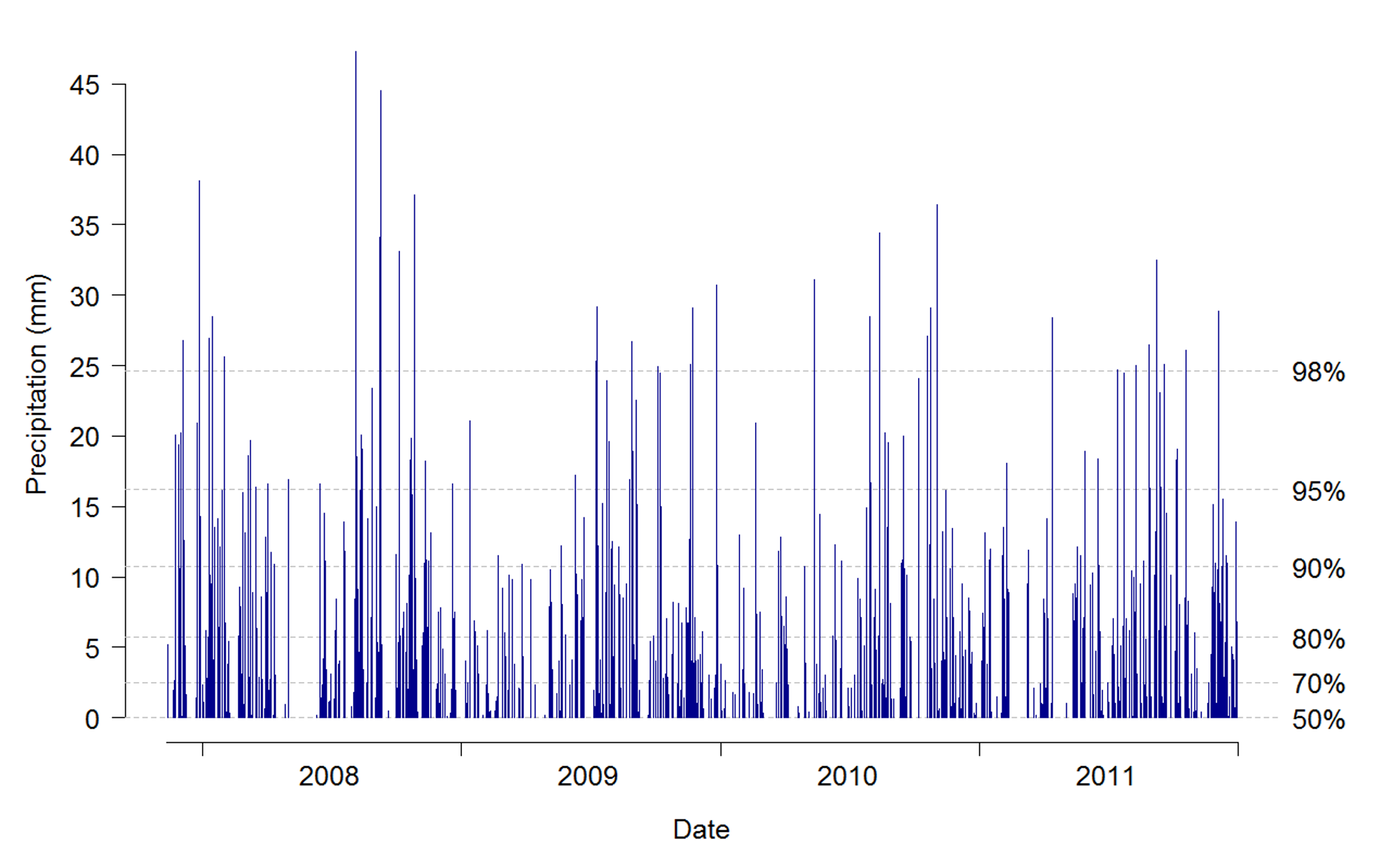

Supplement: Figure S2 — Precipitation. Daily registered precipitation from November 08, 2007 to December 31, 2011, 30 km upstream of the raw water intake of the Alelyckan drinking water utility. Horizontal lines represent selected percentiles. (TIFF) [file pone.0069918.s002.tif]

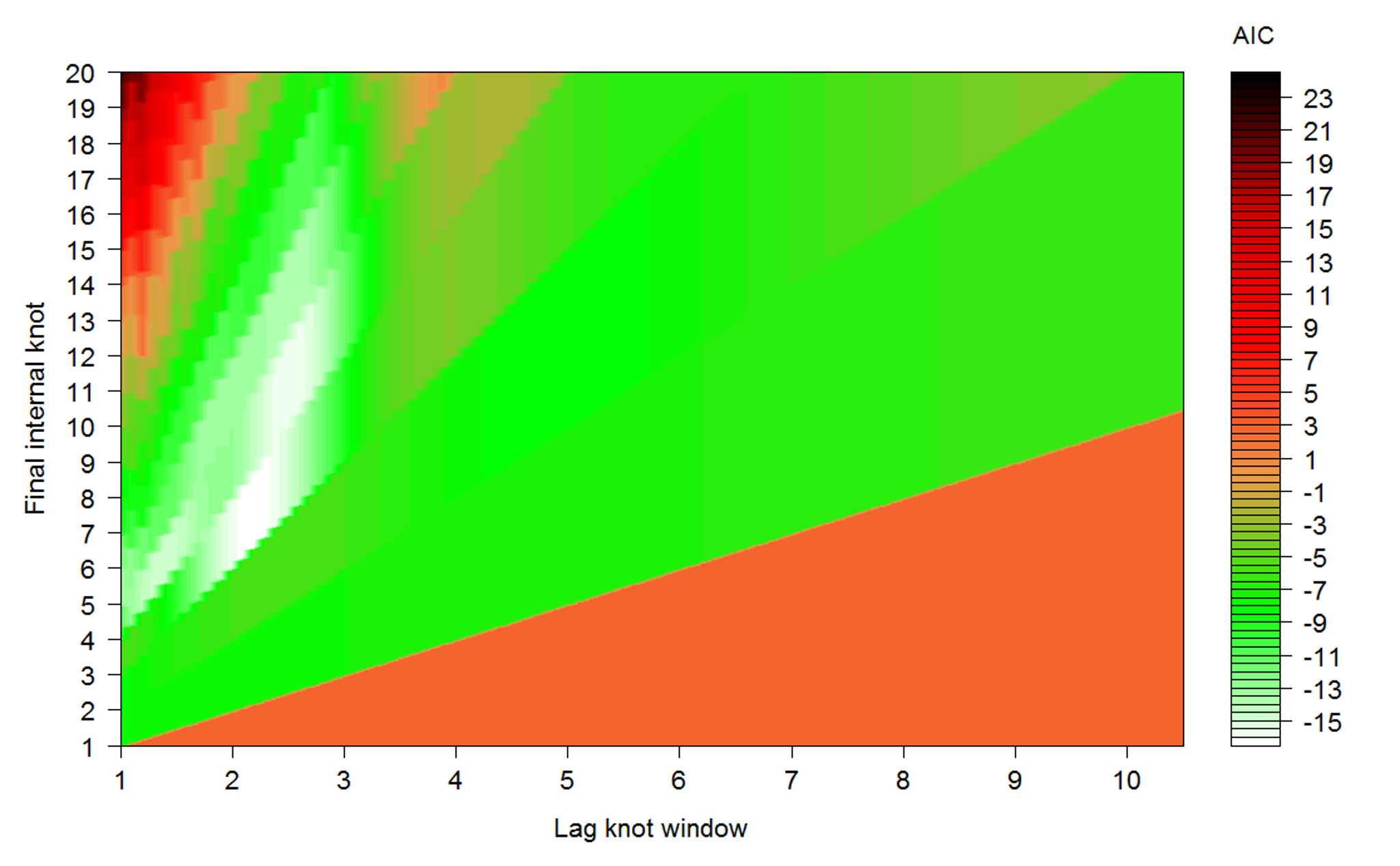

Supplement: Figure S3 — AIC scores. AIC scores from models describing the association between precipitation and GI-calls with different knot settings in lag space in the DLNM predictor, with use of natural cubic spline design and one internal knot in predictor space. Colors represent the change in the AIC score when including the DLNM predictor in the model. Lower triangular area has invalid combinations and represents a model with zero internal lag knots. The AIC suggest that a best lag knot setting is 3 internal lag knots with a lag knot window at 2.2 ({2.2, 4.4, 6.6}). (TIFF) [file pone.0069918.s003.tif]

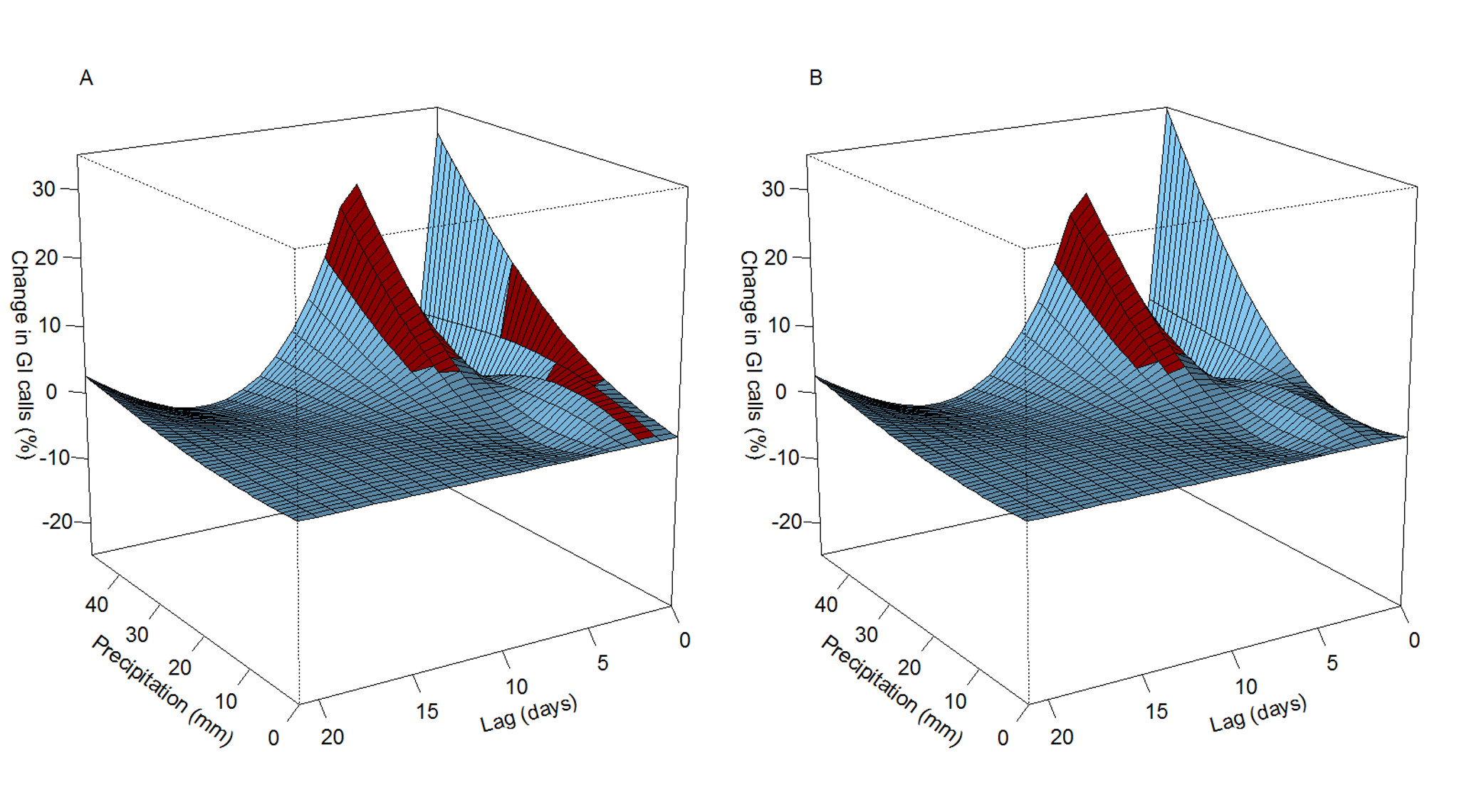

Supplement: Figure S4 — Modifications due to collinearity. Estimated change in nurse advice calls (%) relating to GI symptoms to daily precipitation (0–47 mm) along 0–21 lags with and without adjustment for consecutive dry or wet weather days. Red areas show where significant positive relationships are estimated in at least 2 consecutive days. A: a distributed non-linear lag model not adjusted for consecutive days with dry or wet weather. B: a distributed non-linear lag model adjusted for consecutive days of wet weather. (TIFF) [file pone.0069918.s004.tif]

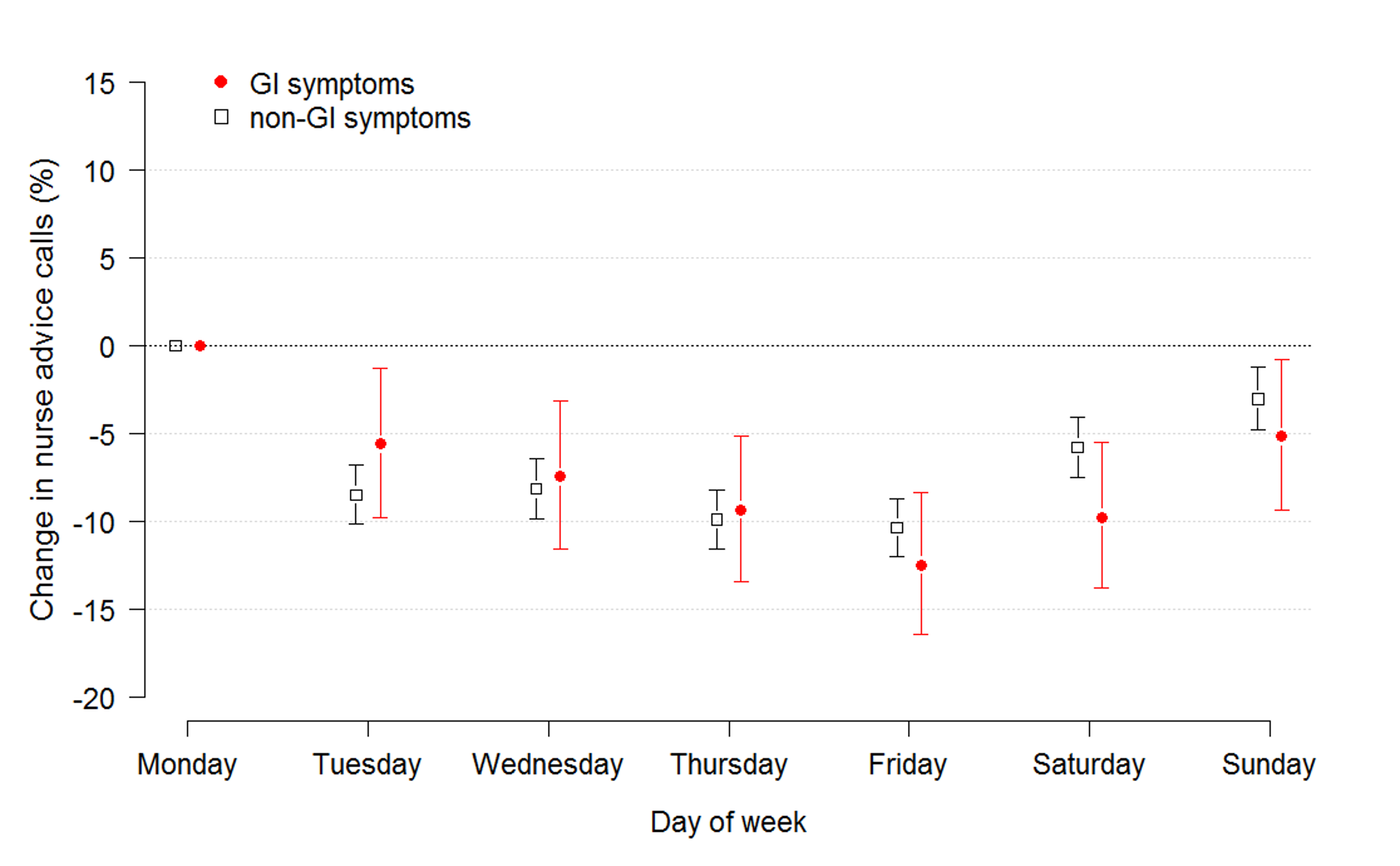

Supplement: Figure S5 — Day of week effect. Effect of day of week on nurse advice calls where Mondays is selected as reference day. (TIFF) [file pone.0069918.s005.tif]

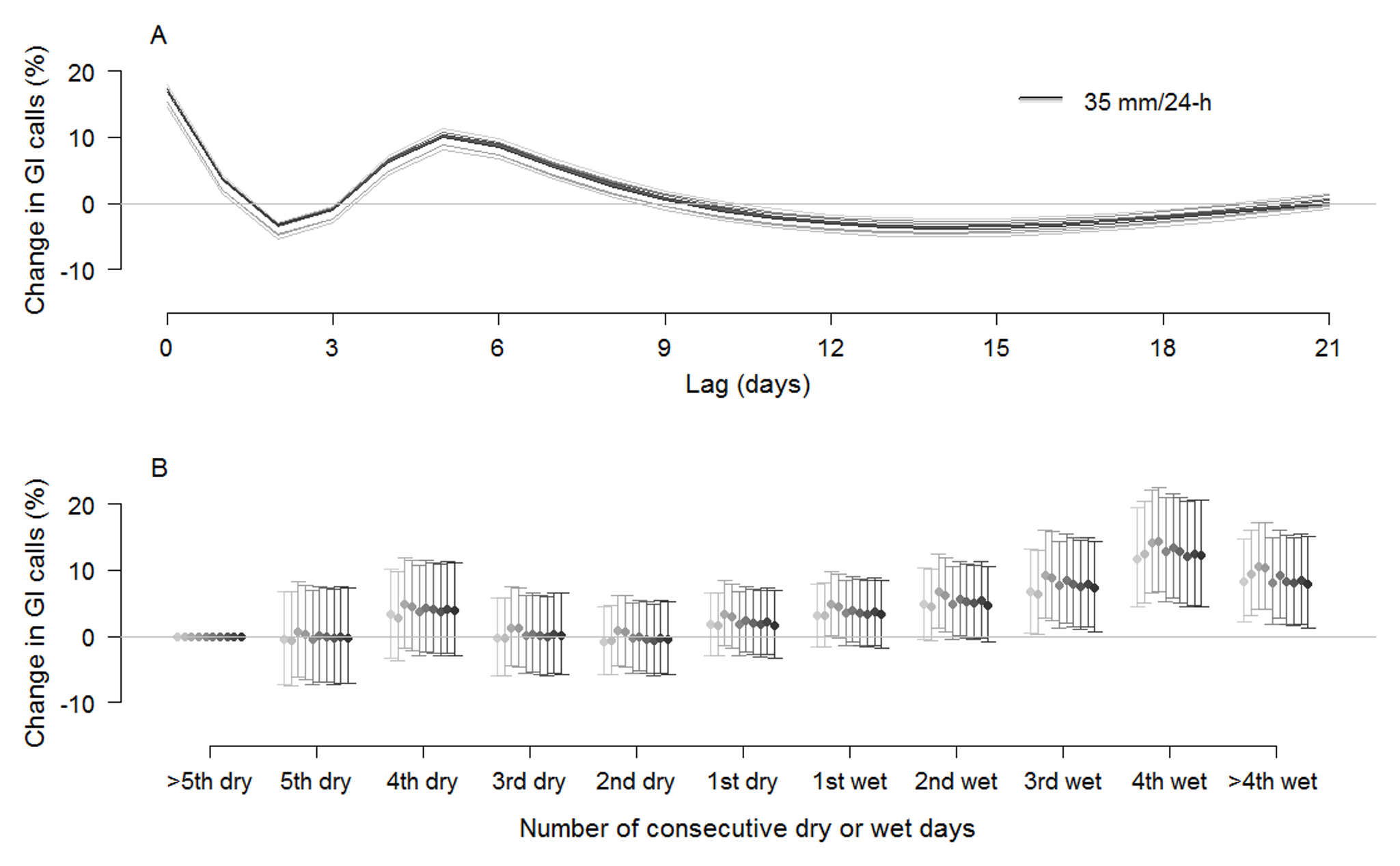

Supplement: Figure S6 — Sensitivity analyzes. Association between precipitation and GI calls with different settings (df) in the seasonal- trend component (10 models). Light gray (3 df per year) – dark gray (12 df per year). A: estimated change (%) in GI calls of an event of 35 mm/24-h of precipitation along 0–21 lag days. B: estimated change (%) in GI calls with consecutive dry or wet weather days. (TIFF) [file pone.0069918.s006.tif]

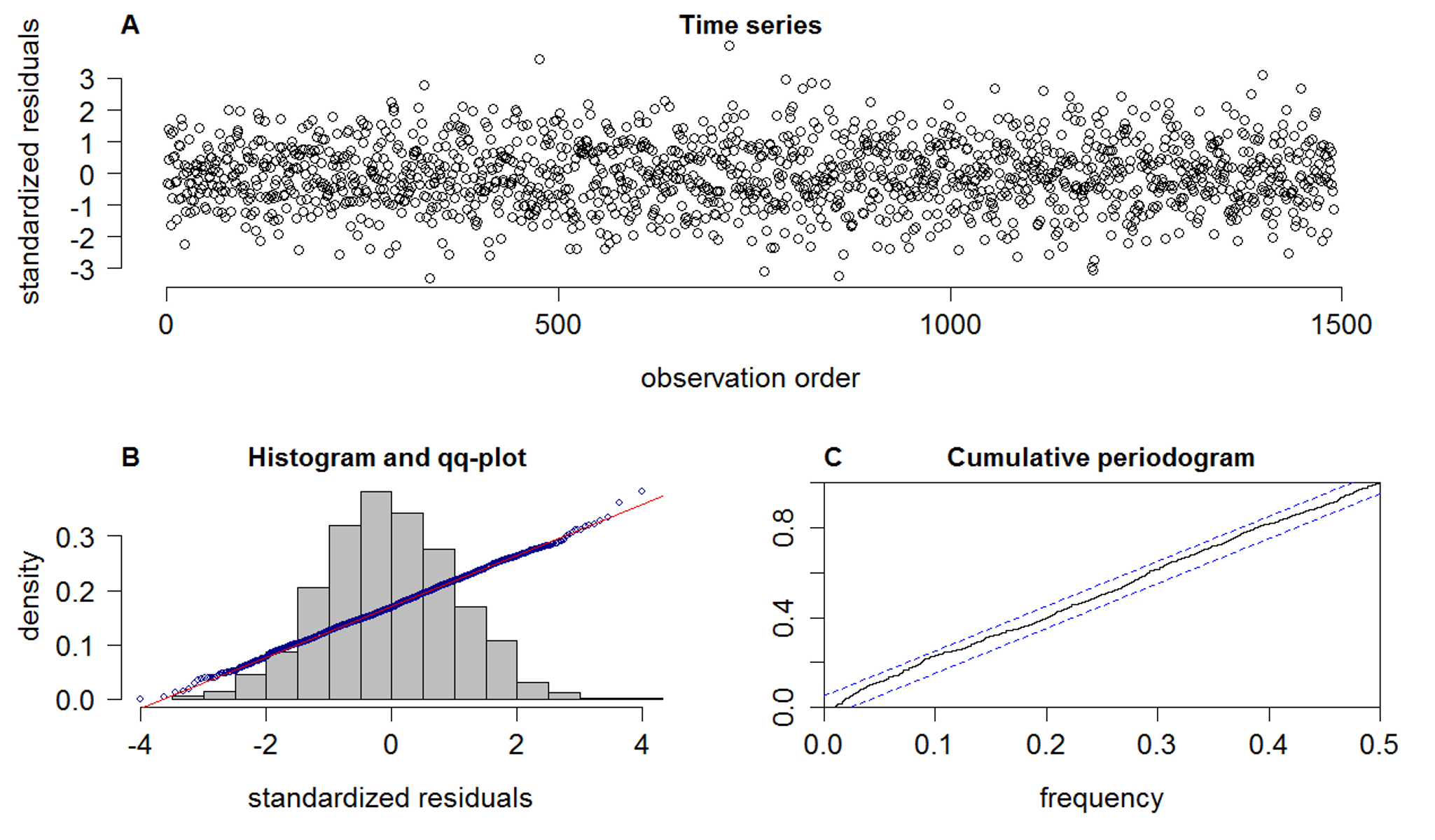

Supplement: Figure S7 — Model residuals. Diagnostic plots of model residuals (DLNM model with trend/season component using 7 df per year). A: time series residual plot indicating constant variance with time a no seasonal pattern. B: histogram and qq-plot show that residuals follow a normal distribution. C: the cumulative periodogram indicate no serial correlation in model residuals. (TIFF) [file pone.0069918.s007.tif]
